# Wiedemann-Steiner syndrome with a novel pathogenic variant in KMT2A: a case report

**DOI:** 10.25100/cm.v50i1.3555

**Published:** 2019-03-30

**Authors:** Diana Ramirez-Montaño, Harry Pachajoa

**Affiliations:** 1 Universidad Icesi, Facultad de Ciencias de la Salud. Centro de Investigaciones en Anomalías Congénitas y Enfermedades Raras (CIACER). Cali, Colombia.; 2 Fundación Clínica Valle del Lili. Cali, Colombia.

**Keywords:** Frameshift mutation, histone methyltransferases, hypertrichosis, intellectual disability, whole exome sequencing, Secuenciación del Exoma completo, Hipertricosis, Mutación frameshift, Metiltransferasa de histonas, Discapacidad intelectual

## Abstract

**Case Description:**

We report the case of a one-year-old girl who was diagnosed with Wiedemann-Steiner Syndrome based on the identification of a novel *de novo* frameshift mutation in the *KMT2A* gene by whole exome sequencing and supported by her clinical features.

**Clinical Findings:**

*KMT2A* mutations cause Wiedemann-Steiner Syndrome, a very rare genetic disorder characterized by congenital hypertrichosis, short stature, intellectual disability, and distinct facial features.

**Treatment and Outcome:**

Whole exome sequencing identified a novel frameshift variant: c. 4177dupA (p.Ile1393Asnfs * 14) in *KMT2A*; this change generates an alteration of the specific binding to non-methylated CpG motifs of the DNA to the protein. The genotype and phenotype of the patient were compared with those of earlier reported patients in the literature.

**Clinical Relevance:**

In diseases with low frequency, it is necessary to establish a genotype-phenotype correlation that allows the establishment of therapeutic and follow-up goals. The phenotype comparation with other reported cases did not show differences attributable to sex or age among patients with Wiedemann-Steiner Syndrome. Whole exome sequencing allows identifying causality in conditions with high clinical and genetic heterogeneity like hypertrichosis.

## Resumen

**Descripción del caso:**

Se reporta el caso de una paciente femenina de un año de edad, diagnosticada con Síndrome de Wiedemann-Steiner basado en la identificación de una nueva variante patogénica *de novo* de tipo frameshift en el gen *KMT2A* Mediante secuenciación de exoma usando el enfoque de trio, sumado a sus características clínicas.

**Hallazgos clínicos:**

las mutaciones en *KMT2A* causan el Síndrome de Wiedemann-Steiner, un desorden genético muy raro caracterizado por hipertricosis congénita, talla baja, retardo mental variable y fenotipo facial distintivo, los cuales se encuentran en la paciente reportada.

**Resultado:**

La Secuenciación de exoma completo encontró una variante de tipo frameshift: c.4177dupA (p. Ile1393Asn*fs* * 14) en *KMT2A,* este cambio a nivel génico genera una alteración de la unión específica a motivos CpG no metilados del DNA a la proteína. El genotipo y el fenotipo de la paciente fue comparado con los pacientes reportados previamente en la literatura.

**Relevancia clínica:**

En enfermedades con baja frecuencia como la aquí reportada es necesario establecer correlaciones genotipo-fenotipo que permitan establecer planes terapéuticos y de seguimiento. El análisis realizado no evidenció diferencias atribuibles a sexo o edad entre los pacientes diagnosticados con Síndrome de Weidemann-Steiner. La secuenciación de exoma permitió identificar causalidad en este caso, cuya característica principal de hipertricosis se asocia con alta heterogeneidad clínica y genética.

## Introduction

The genetic basis of congenital hypertrichosis is still unknown. Non-androgenic excessive growth of terminal hair is associated with several rare genetic conditions ^1^ ^,^ ^2^. One of them is the Wiedemann-Steiner Syndrome (WDSTS) (MIM # 605130), a rare autosomal dominant disorder described for the first time by Wiedemann *et al*. ^3^, and defined as a syndrome in 2000 by Steiner et al ^4^. This syndrome is characterized by deficiency of pre and postnatal growth, hypertrichosis cubiti or generalized hypertrichosis, psychomotor delay, intellectual disability with behavioral alterations and distinctive facial features with narrow nose, sinofris, ocular hypertelorism, long philtrum, short palpebral fissures, low set ears and an ogival palate. Other associated physical findings include dilation of the renal calyces, convergent strabismus and limb shortening ^3^ ^,^ ^4^. To date, 26 patients with WDSTS have been reported in the global literature ^1^ ^,^ ^5^ ^,^ ^6^, and only one of them is from Latin America ^7^. Clinical overlap between WDSTS and other genetic syndromes with hypertrichosis such as Kabuki Syndrome (KS, MIM # 147920, 300867), Coffin-Siris Syndrome (CS, MIM # 135900), Pierpont Syndrome (PS, MIM # 602342) and Cornelia de Lange Syndrome (CdLS, MIM # 122470) ^2^ may complicate the diagnosis.

In 2012, Jones *et al*. ^1^ performed whole exome sequencing in six patients with hypertrichosis and clinical features of WDSTS and identified novel and *de novo* dominant mutations in the *KMT2A gene* (lysine methyltransferase 2A, known previously as MLL) in five of them. They concluded that haploinsufficiency and heterozygous mutations in this gene were the genetic causes of WDSTS. No differences were found for gender. The 49 pathogenic variants reported to date in LOVD 3.0 for the *KMT2A* gene (https://databases.lovd.nl/shared/variants/KMT2A) correspond mostly to mutations that lead to prematurely truncated proteins ^1^ ^,^ ^5^ ^,^ ^6^. These pathogenic variants in *KMT2A* are associated with defects in chromatin remodeling and, consequently, in the regulation of gene expression ^8^ ^,^ ^9^.

At this stage, the complete phenotype of WDSTS is not understood fully. We report the case of a girl from Colombia with clinical features of WDSTS in whom we identified a not previously reported pathogenic variant of *KMT2A*. To establish a genotype-phenotype correlation as the major aim of this report, her clinical features are compared with previously reported WDSTS patients.

## Case Description

We describe the case of a 21-month-old female patient from southwest Colombia, who was the second child of a 34-year-old mother and a nonconsanguineous 36-year-old father, both without a significant family history. The mother’s pregnancy was uncomplicated, and prenatal ultrasounds were normal. A cesarean delivery was performed at 38 weeks because of the breech position of the baby. The birth weight was 3,324 g (48^th^ centile). The baby showed spontaneous neonatal adaptation with APGAR 9 and 10 at 1 and 5 minutes, respectively. She was released jointly with her mother on the second day after birth.

At three months of age, she was assessed by a neuropediatrics service for generalized hypotonia associated with psychomotor development delay. At six months of age, a low weight and height were documented as well as generalized hypertrichosis. The occurrence of this neurological symptoms together with persistent hypertrichosis at 12 months led to an assessment by a pediatric endocrinologist, who ruled out an androgenic hormone disorder (normal testosterone levels, α-OH-progesterone and somatomedin). At that age, she was also assessed by a pediatric gastroenterologist who diagnosed moderate gastroesophageal reflux that required pharmacological management. Later, at 20 months of age, she presented with two episodes of urinary infection, one of them complicated by pyelonephritis. 

Regarding her development, she achieved cephalic support at 12 months, and assisted sitting at 18 months. At the age of 21 months, she did not exhibit age-appropriate language development.

The paraclinical tests performed on the patient included Normal brain MRI performed at 10 months of age. A renal ultrasound, dimercapto succinic acid renal scan and voiding cystourethrography were performed at 20 months of age and were reported to be normal. Other studies performed at this time were karyotype, blood and urine metabolic screening, creatinine phosphokinase, complete blood count, fasting glucose test, transthoracic echocardiogram, auditory and visual evoked potentials; all of them were reported as normal. X-rays of the extremities performed at 21 months of age showed bilateral congenital hip dislocation.

**Figure 1:**
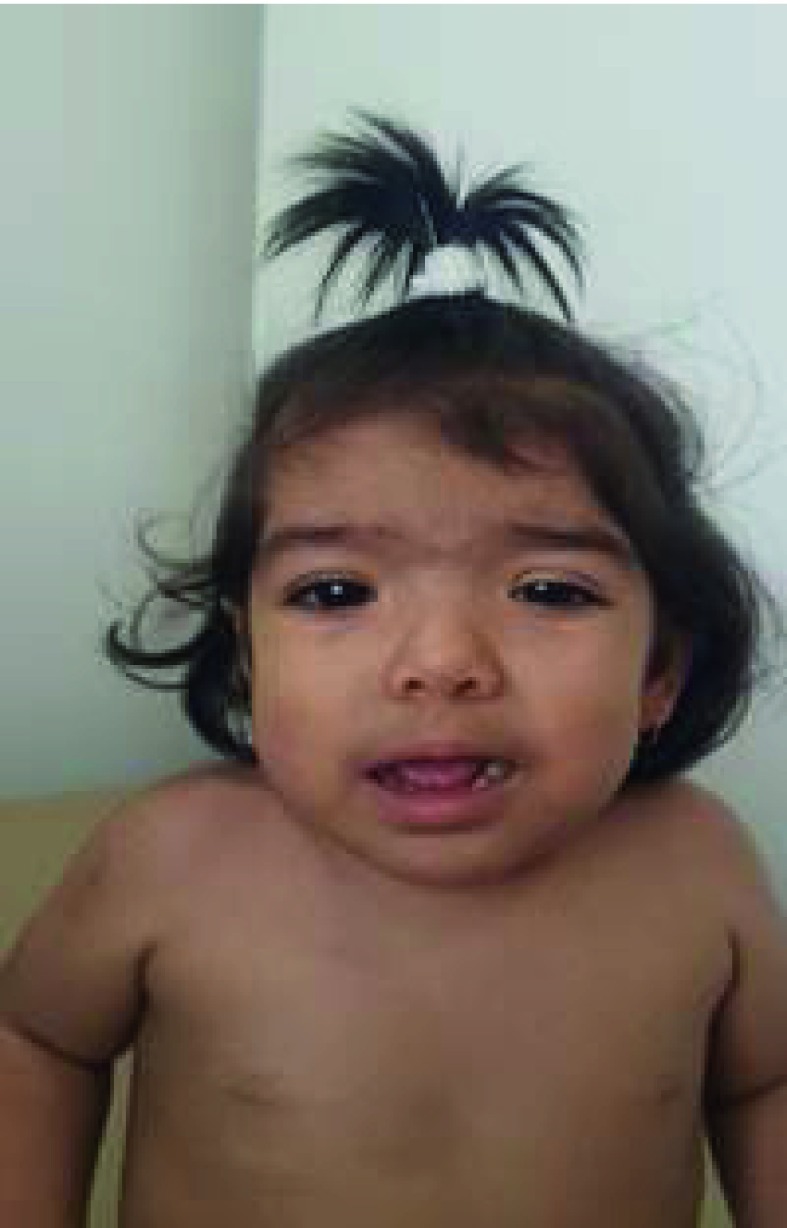
Facial appearance of one-year-old girl with Wiedemann-Steiner Syndrome**.** frontal hypertrichosis, low anterior hairline, thick eyebrows, synophrys, long eyelashes, hypertelorism, left palpebral ptosis, epicanthic folds, downslanted palpebral fissures, low set ears, and wide and depressed nasal bridge.

**Figure 2:**
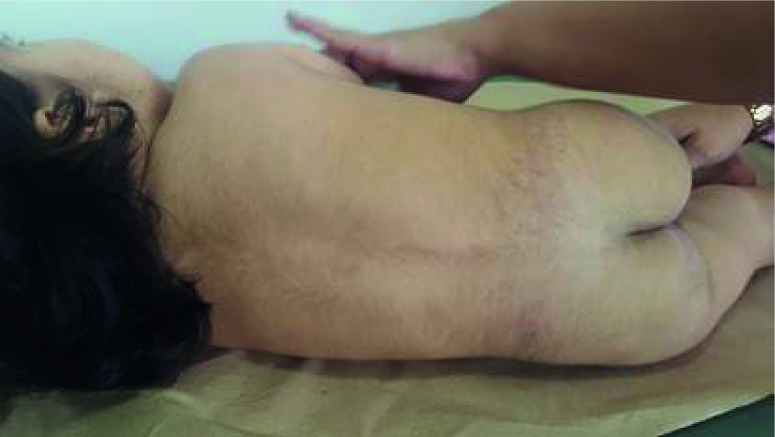
Physical appearance of one-year-old girl with Wiedemann-Steiner Syndrome: generalized hypertrichosis predominantly around the midline and low back region.

At 21 months of age, she was referred for genetic assessment because of delayed psychomotor development, generalized hypotonia, low height, and hypertrichosis. Her weight was 8,7 kg (-2,2 SD) and her height was 72 cm (-3,83 SD). Physical examination revealed round facies, thick eyebrows, synophrys, long eyelashes, downslanted palpebral fissures, hypertelorism, long philtrum, Dennis Morgan folds, and excessive thick facial hair mainly in the frontal region (Fig. 1). Generalized hypertrichosis was present and more pronounced on the back and around the mammillae (Fig. 2). Other findings included mild generalized hypotonia, broad feet, and irritability without hyperactivity. 

Further investigation was performed using whole exome sequencing (WES) in the trio approach with a massive sequencing platform (CeGaT-GmbH, Tübingen, Germany). WES was performed on the sequencing coding and flanking intronic regions using the HiSeq2500/4000 system (Illumina®, San Diego, CA, US). The CASAVA 1.8 analysis package (Illumina®, San Diego, CA, US) was used to demultiplex the sequencing reads. The trimmed reads were mapped to the human reference genome (GRCh38) using the Burrows-Wheeler aligner software. A novel frameshift pathogenic variant in the heterozygous state of the *KMT2A* gene (v1. NM_001197104.1) was identified: c. 4177dupA (p. Ile1393Asnfs*14). This variant was not identified in either of the patient’s parents and has not been reported previously in population databases. This variant generates a change in the reading frame that results in the premature truncation of the protein or degradation of the messenger RNA. This finding was confirmed by Sanger sequencing and was compatible with the diagnosis of WDSTS. No other gene variants were identified in this case.

According to the American College of Medical Genetics and Genomics (ACMG) Guidelines for the Interpretation of Sequence Variants ^10^, this variant is classified as pathogenic (PVS1, PS2, PM2, and PP3 criteria). The variant functional prediction software tools SIFT (https://sift.bii.a-star.edu.sg/), Functional Analysis through Hidden Markov Models FATHMM (http://fathmm.biocompute.org.uk/) and Polymorphism Phenotyping v2 (Polyphen-2 http: // genetics. bwh.harvard.edu/pph2/) classified it as a deleterious/damaging variant (disease causing) because of its high evolutionary conservation.

The *de novo* inheritance of the mutation was explained to her parents, as was the recurrence risk that varies from 3% to 5% in future pregnancies ^11^. The patient's follow-up plan includes annual renal and cardiac tests to assess other syndrome-associated features that may not yet be present in the patient due to the young age of the diagnosis. Neurological follow-up includes a therapy intervention for the hypotonia and the possible intellectual commitment.

The patient’s parents provided written informed consent for the publication of her case report and accompanying images.

## Discussion 

Exome sequencing has revolutionized the genetic study of monogenic diseases over the last decade. This diagnostic tool allows the time- and cost-effective sequencing of the whole exome (approx. 3% of the genome) to identify genetic causes of dysmorphological syndromes with high clinical and genetic heterogeneity. Exome sequencing has improved diagnostic performance in medical genetic practice by more than 25%, leading to a better understanding of molecular mechanisms involved in pathologies with Mendelian inheritance ^*12*^. This method has enabled the identification of new genes responsible for unclear cases that had remained without diagnosis previously, and of new syndromes involved in human disease ^*12*^.

WDSTS is one of the new syndromes identified by exome sequencing since 2012 ^*1*^. Its clinical features overlap with other syndromes that present with hypertrichosis ^*13*^. Pathogenic variants in the *KMT2A* gene have been identified as a cause of this syndrome in studies with large sample sizes that validate the clinical utility of next-generation sequencing tools ^*14*^ ^,^ ^*15*^. The recurrent appearance of recently characterized genes in these studies is probably due to the previous unavailability of clinical tests for gene analysis either individually or as part of a multigenic panel that included the new loci associated with WDSTS ^*14*^. 

It is challenging to diagnose WDSTS because the phenotype shows extensive variation and is not defined clearly. In the young female patient reported here, the facial features appear to be similar to patients reported in other studies. Synophrys, long eyelashes, ocular hypertelorism and long philtrum ^*1*^ ^,^ ^*5*^ are characteristic for all of them. While they may appear as early as one year of age, they become accentuated with age ^*16*^. Other features include generalized hypertrichosis that has been reported in more than 80% of WDSTS patients, and was prominent at the back in our case ^*1*^ ^,^ ^*5*^ ^,^ ^*6*^. Hypertrichosis cubiti is frequently associated with this syndrome and is considered the most prominent feature, but was not present in our patient. Prenatal growth retardation reported by other authors ^*1*^ ^,^ ^*3*^ ^,^ ^*5*^ ^,^ ^*6*^ and microcephaly, seen in 50% of patients with WDSTS, were not present either ^*5*^. Table 1 summarizes the phenotypic characteristics of our patient as compared to what is described in the literature for other patients with WDSTS.

**Table 1:** Summary of phenotypic features in 1-year old female patient compared to features present in more than 60% of patients with WDSTS.

Clinical features present in patient in this case report	% of patients with WDSTS that exhibit the feature
Postnatal growth retardation	100
Psychomotor development delay	100
Intellectual disability (variable)	100
Depressed nasal bridge	100
Bulbous nose	100
Long eyelashes	94.7
Wide nasal bridge	89.5
Downslanted palpebral fissures	88.9
Thick hair	85.7
Prominent digital pads	83.3
Dorsal hypertrichosis	83.3
Low anterior hairline	75.0
Broad nasal tip	73.7
Thick eyebrows	71.4
Hypertelorism	66.7
Hypotonia	66.7
Palpebral ptosis	63.6
Clinical features not present in patient in this case report	% of patients with WDSTS that exhibit the feature
High palate	88.3
Prenatal growth retardation	66.7
Short columela	66.7
Macroglossia	66.7
Single transverse palmar crease	66.7
Small hands and feets	63.6
Clinodactyly	60.0

Neurological manifestations reported in WDSTS include intellectual disability and psychomotor development delay that are present in 100% of patients, and hypotonia, which can be found in 66.7%. Both of these features were present in our patient. She also exhibited a speech and language developmental delay. Because of her age, an IQ test was not performed. Irritability is a prominent feature in the behavior of WDSTS patients and has been reported in about 20% in combination with hyperactivity, heteroagression, and autistic features ^17^. Our patient also presented congenital bilateral hip dislocation that has been reported in 34% of patients with WDSTS ^11^. Other features that were reported previously but were ruled out in our patient are feeding problems (58%), urinary system anomalies (34%) and cardiac abnormalities (31.3%) associated with high morbidity ^1^ ^,^ ^5^ ^,^ ^17^ ^,^ ^18^.

The *KMT2A* gene encodes a histone methyltransferase related to the regulation of gene expression. This protein plays an essential role in the DNA packaging for early development and hematopoiesis, mediating chromatin modifications associated with epigenetic transcriptional activation. The *KMT2A* gene shows broad expression in almost all human tissues ^14^ ^,^ ^15^ and contains 36 exons that encode different isoforms (Ref Seq 2010). The mutations reported in WDSTS so far have all been *de novo*, meaning a new variant is reported with each clinical report. The majority of mutations are nonsense, frameshift, splice site deletion, or exon deletion, leading to a truncated transcript (messenger RNA) or protein. The mutation reported here c.4177dupA (p.Ile1393Asnfs * 14) is not annotated in the 1,000 Genomes Project, ExAC, or EVSor in-house database, and, so far, has not been reported in the literature. Frameshift type mutations, such as this one, constitute very strong pathogenic criteria according to the ACMG classification of sequencing variants, because they modify the length of the protein by inserting premature stop codons with the shift of the reading frame; therefore, it is usually related to severe phenotypes in monogenic diseases ^10^.

**Figure 3:**
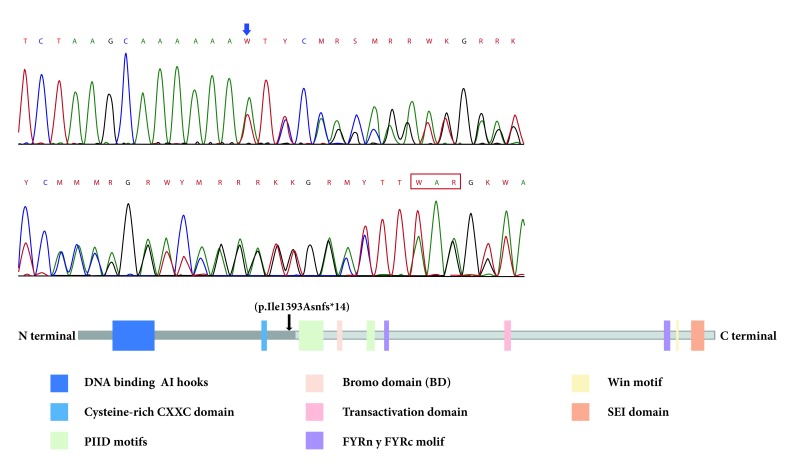
A. Location of reported KMT2A mutation: Electrophogram of exon 9 of the *KMT2A* gene showed duplication of adenine (blue arrow) at position 4177 **c.**4177dupA. This has a frameshift effect, generating a premature stop at codon 13 positions later (red box). B. Position at the protein level: The mutation leads to premature termination of the translation before the c-terminal region (black arrow) that contains the BD domain, transactivation domain and SET domain, as PHD motifs that are essential for gene recognition, transactivation and transformation associated with protein function. FRYN, FYRC, and Win motifs are not expressed either.

The pathogenic variants in the *KMT2A* gene that cause WDSTS are distributed throughout the gene, but located before the FYRC domain. This domain is important for the recognition of a protein after cleavage. More than 50% of reported mutations have been in exons 3 and 27, the two longest exons of this gene; however, mutational hot-spot sites have not been identified ^5^. Our patient showed a pathogenic variant in exon 9, between two conserved domains of this protein, the cysteine-rich CXXC, homologous to the DNA metil transferase domain, and the Plant Homeodomain finger (PHD) motif (Fig. 3). Both these domains allow specific binding to non-methylated CpG DNA motifs, essential for gene recognition, transactivation and transformation associated with MLL proteins ^19^. There is only one patient with a similar mutation, who was reported by Mendelsohn et al ^20^. Their patient had an intragenic deletion of exons 2-10 (Table 2). This patient had recurrent urinary tract infections and renal alterations. Our patient also had two episodes of urinary tract infections, one of them complicated by pyelonephritis at the age of 20 months. A nephrology assessment ruled out renal alteration; however, it is a current recommendation to perform regular renal ultrasound in the follow-up of patients with WDSTS ^6^. The heterozygous state of the variant with autosomal dominant inheritance acts like a null allele that leads to a prematurely truncated protein interfering with the normal protein. This is similar to what is reported in the literature when a null or missing allele have a negative dominant effect over the normal allele ^6^.

**Table 2:** Summary of mutations in the *KMT2A* gene in patients with WDSTS in the literature.

Patients	Amino acid change	Affected gene region	Ethnicity	Author
WDST1	p.V2936*	Exon 27	Not reported	Jones et al. ^1^
WDST2	p.L2756*	Exon 27		
WDST3	p.S2305Lfs*2	Exon 27		
WDST5	p.R2382*	Exon 27		
WDST6	p.K1534*	Exon 13		
Patient 1	p.C1448R	Exon 11	Mexican	Strom et al. ^7^
Patient 2	c.4086+1G>A	Intron 8	Caucasian	
	chr11:118,339,487-118,355,089del	Exon 2 to 10	Hispanic	Mendelson et al. ^20^
P1	p.G2422*	Exon 27	Not reported	Zemojtel et al. ^21^
P8	p.E3448fs*7	Exon 27		
	p.R2127*	Exon 26	Not reported	Calvel et al. ^22^
Twin 1	p.R1083*	Exon 4	Caucasian	Dunkerton et al. ^18^
Twin 2	p.R1083*	Exon 4		
	p.R1636*	Exon 15	Arabian	Steel et al. ^23^
Patient 1	p.R2480*	Exon 27	Japanese	Miyake et al. ^24^
Patient 2	p.Q2261*	Exon 27	Japanese	
Patient 3	p.C1189Y	Exon 5	Australian	
Patient 4	p.Pro280Thr	Exon 3	Japanese	
Patient 5	p.V347Lfs*53	Exon 3	Japanese	
Patient 6	p.L717Cfs*39	Exon 3	Japanese	
CdLS 3	p.R745*	Exon 3	Turkish	Yuan et al. ^25^
	p.C1161G	Exon 5	Italian	Stellacci et al. ^26^
A.II-5	p.Q2803*	Exon 27	Chinese	Sun et al. ^5^
B.II-1	p.Q819*	Exon 3	Chinese	
	p. Pro51Argfs*84	Exon 1	Not reported	Argawall et al. ^27^
	p. Ile1393Asnfs*14	Exon 9	Colombian	This report

In summary, we reported the first case of a patient from Colombia with a frameshift pathogenic variant not reported previously in the *KMT2A* gene. The phenotype was similar to what is reported in the worldwide literature. This 1-year-old patient had a clinical history of urinary infection episodes without renal impairment or pathological findings in imaging, which seems to be related to the methyltransferase activity of the protein located in the domains and motifs affected by the mutation as described here. 

This report is an approach to a possible genotype-phenotype correlation for WDSTS, in which, in case of hypertrichosis, neurological and renal involvement must always be ruled out. The most prominent features of WDSTS that can serve as diagnostic criteria are generalized hypertrichosis, postnatal growth retardation, psychomotor development delay and distinct facial phenotype (thick hair and eyebrows, hypertelorism, downslanted palpebral fissures, and long eyelashes). In more than 70% of the patients reported in the literature, these features are present.
