# Supplementary material for: Wiedemann-Steiner syndrome with a novel pathogenic variant in KMT2A: a case report
Source: Colomb Med (Cali). 2019 Mar 30;50(1):40–5. doi: 10.25100/cm.v50i1.3555 (PMC6536042; doi:10.25100/cm.v50i1.3555)
Supplement: Related texts

# Case report: Síndrome de Wiedemann-Steiner por una nueva variante patogénica en KMT2A: reporte de un caso

## Introducción

Las bases genéticas de la hipertricosis congénita aún son desconocidas, el crecimiento excesivo del vello terminal no relacionado con andrógenos se asocia a varias condiciones genéticas raros ^1^^,^^2^. Uno de ellos es el síndrome de Wiedemann-Steiner (SWS) (MIM #605130), un trastorno autosómico dominante raro descrito por primera vez por Wiedemann et al.^3^, y definido como síndrome en el 2000 por Steiner *et al*^4^. Este síndrome es caracterizado por deficiencia de crecimiento pre y postnatal, hipertricosis cubiti o generalizada, retraso psicomotor, discapacidad intelectual con alteraciones del comportamiento y facies características con nariz estrecha, sinofris, hipertelorismo ocular, filtrum largo, fisuras palpebrales cortas, orejas de implantación baja y paladar ojival. Otros hallazgos asociados incluyen dilatación de calices renales, estrabismo convergente y acortamiento de extremidades ^3^^,^^4^. Se han reportado hasta el momento 26 pacientes en la literatura global con características del SWS ^1^^,^^5^^,^^6^, de los cuales solo un paciente es originario de américa Latina ^7^. Las características de este síndrome se sobrelapan con otros síndromes asociados a Hipertricosis como Kabuki Syndrome (KS, MIM 147920, 300867), Coffin Siris (CS, MIM 135900), Pierpont Syndrome (PS, MIM 602342) y Cornelia de Lange syndrome (CdLS, MIM 122470) ^2^ lo que puede dificultar su diagnóstico.

En el año 2012, Jones *et al*.^1^, realizaron secuenciación exómica (*Whole exome secuencing*) de pacientes con características clínicas del SWS identificando mutaciones en estado heterocigoto *de novo* en el gen *KMT2A* (antes conocido como MLL) en cinco de seis pacientes y concluyendo que la haploinsuficiencia de este gen era la causa genética de esta entidad . Hasta el momento no se han encontrado diferencias en género, las 49 variantes patogénicas reportadas hasta el momento en LOVD 3.0 para el gen (https://databases.lovd.nl/shared/variants/KMT2A) corresponden en su mayoría a mutaciones *de novo* que generalmente conducen a proteínas truncadas prematuramente ^1^^,^^5^^,^^6^. Estas variantes en *KMT2A* generan defectos en la remodelación de la cromatina que participa en la regulación de la expresión génica ^8^^,^^9^.

El fenotipo completo del SWS no se entiende completamente. Se presenta una paciente de origen colombiano con esta entidad, quien además presenta una variante patogénica no reportada en *KMT2A,* y se comparan sus características clínicas con los pacientes reportados previamente tratando de establecer una correlación genotipo-fenotipo.

## Reporte de caso

Paciente femenina de 21 meses de edad, producto de la segunda gestación de la madre. La madre con 34 años y el padre con 36 años no consanguíneos. No hay historia familiar significativa. Embarazo normal con ultrasonidos prenatales normales. Parto por cesárea a las 38 semanas de gestación, debido a presentación podálica del feto. Peso al nacer: 3,324 g (Percentil 48), adaptación neonatal espontanea con APGAR 9-10 al 1 y 5 minutos, respectivamente. Fue dada de alta conjunta con su madre al segundo día de nacida.

La niña fue valorada al tercer mes de edad por neuropediatría por el cuadro clínico de hipotonía generalizada, asociado a un retraso en el desarrollo psicomotor. Desde los 6 meses de edad se documentaron un bajo peso y talla. Debido a la asociación del cuadro neurológico con hipertricosis, fue valorada a la edad de 12 meses, por endocrinología pediátrica quienes descartaron un trastorno hormonal de tipo androgénico (Niveles de testosterona, α-OH-progesterona y Somatomedina normales). A esa misma edad fue valorada por gastroenterología y nutrición debido al bajo peso quienes diagnosticaron un reflujo gastroesofágico moderado que requirió un manejo farmacológico. Adicionalmente, a la edad de 20 meses, presentó dos episodios de infección urinaria, uno de ellos complicado con pielonefritis. El desarrollo psicomotor de la paciente fue retrasado, logró el sostén cefálico a los 12 meses, sedestación asistida a los 18 meses y hasta la edad de 21 meses no había un desarrollo del lenguaje. 

Los paraclínicos realizados incluyen: Resonancia magnética cerebral realizada a los 10 meses de edad con resultado normal. Ecografía renal, DMSA y Cistografía miccional realizadas a los 20 meses, normales. Otros estudios realizados a la edad de 20 meses fueron Cariotipo, tamizaje metabólico en sangre y orina, CPK, hemograma, glicemia en ayunas, ecocardiograma, potenciales evocados auditivos y visuales, todos reportados como normales. Las radiografías de extremidades realizadas a la misma edad evidenciaron luxación congénita bilateral de la cadera.

Fue referida para evaluación genética debido a la historia del retraso del desarrollo, hipotonía y baja talla. Al momento de la valoración, a los 21 meses de edad, el peso fue de 8.7 kg (-2.2 DE), altura 72 cm (-3.83 DE) y circunferencia de la cabeza de 47.2 cm (Percentil 30 -0.5 DE). El examen físico evidenció facies redondas, cejas gruesas, sinofris, pestañas largas, fisuras palpebrales inclinadas hacia abajo, hipertelorismo ocular, filtrum largo, pliegues de Dennis Morgan y excesivo vello facial grueso principalmente en región frontal (Fig. 1). Hipertricosis en dorso y región mamilar (Fig. 2). Hipotonía generalizada leve, pies anchos. A nivel comportamental se manifiesta con irritabilidad persistente, no hiperactividad.

**Figura 1:** Apariencia facial. Características evidentes en una vista de perfil frontal a la edad de un año: hipertricosis frontal, línea de implantación anterior del cabello baja, cejas gruesas, sinofris, pestañas largas, hipertelorismo, ptosis izquierda, pliegues epicánticos, fisuras palpebrales inclinadas hacia abajo, orejas de implantación baja y puente nasal ancho y deprimido.

**Figura 2:** Apariencia corporal. Hipertricosis generalizada, más prominente a nivel de línea media y región dorsal baja.

Se realizó el estudio parte de genética de secuenciación exómica utilizando el enfoque de trío (CeGaT-GmbH, https://www.cegat.de/en/). Las regiones codificantes fueron enriquecidas usando la tecnología en solución de Agilent y fueron secuenciadas usando el sistema HiSeq2500/4000 de Illumina. Los datos de salida de Illumina HiSeq se convirtieron de un archivo bcl a un archivo FastQ por el software Illumina CASAVA 1.8, y fueron asignados por el programa Burrows Wheeler Aligner a la secuencia haploide del genoma humano de referencia (GRCh38 https://www.ncbi.nlm.nih.gov/assembly/GCF_000001405.38). *Whole exome sequencing* (WES) reveló una nueva variante patogénica de novo de tipo frameshift: c. 4177dupA (p. Ile1393Asn*fs* * 14) en *KMT2A* (v1. NM_001197104.1), la cual no fue identificada en ninguno de los padres y no se encuentra reportada en las bases de datos poblacionales. Esta variante genera un cambio en el marco de lectura que resulta en el truncamiento prematuro de la proteína o degradación del ARN mensajero, y fue confirmada mediante secuenciación Sanger. Esta variante es compatible con el diagnóstico de Síndrome de Wiedemann-Steiner. No se identificaron más variantes en este estudio. 

Basado en la *ACMG variant interpretation guideline*^10^ la variante se clasifica como patogénica (Criterios PVS1, PS2, PM2 y PP3). Los softwares bioinformáticas de predicción de variantes SIFT (http://sift.jcvi.org/), FATHMM (http://fathmm.biocompute.org.uk/) y Polymorphism Phenotyping v2 (Polyphen-2 http://genetics.bwh.harvard.edu/pph2/) la clasifican con efecto deletéreo dada la alta conservación de la posición en vertebrados. 

Se explicó a la familia la herencia *de novo* de la mutación que implica un riesgo de recurrencia que oscila entre el 3 al 5% en otra gestación ^11^. El plan de seguimiento de la paciente incluyó estudios renales y cardiacos anuales para valorar otras manifestaciones asociadas a la patología que pueden no estar presentes por la edad del diagnóstico y un seguimiento neurológico por neuropediatria que debe incluir una intervención de la hipotonía y el compromiso intelectual asociados con una terapia integral. 

Se obtuvo el consentimiento informado de los padres de la paciente para la publicación del reporte de caso y las imágenes acompañantes. 

## Discusión 

La secuenciación del exoma ha revolucionado el estudio genético de enfermedades monogénicas en la última década*.* Esta herramienta diagnóstica permite la secuenciación del exoma completo (≈3% del genoma) de manera eficiente en términos de costo y tiempo para la identificación de la causa de síndromes genéticos dismorfológicos con alta heterogeneidad clínica y genética ayudando a comprender los mecanismos moleculares de las patologías con herencia mendeliana ^12^. Este método ha permitido también el descubrimiento de nuevos genes y síndromes involucrados en enfermedades humanas que permanecían en odisea diagnóstica y en la actualidad presenta un rendimiento diagnostico mayor al 25% en el ejercicio de la práctica médica de la genética ^12^. 

Uno de los síndromes nuevos identificados mediante la secuenciación de exoma es el SWS el cual comparte algunas características clínicas solapantes con otros síndromes que cursan con hipertricosis y que se conoce solo desde el 2012 ^1^^,^^13^. El gen *KMT2A* ha aparecido como un candidato causante de este síndrome en estudios con gran tamaño de muestra que validan la utilidad clínica de la secuenciación de nueva generación^14^^,^^15^. El predominio de unos genes recientemente caracterizados de manera recurrente entre los hallazgos genéticos en estos estudios, se debe probablemente a la falta de pruebas clínicas disponibles, ya sea por análisis del gen de manera individual o por un panel multigen, para la mayoría de estos nuevos loci asociados con la enfermedad ^14^.

El fenotipo asociado al SWS es amplio por lo que el diagnóstico es un desafío. En la paciente reportada en nuestro reporte de caso, las características faciales parecen ser similares a los pacientes reportados en otros estudios donde se incluye sinofris, pestañas largas, hipertelorismo ocular y filtrum largo ^1^^,^^5^ las cuales se acentúan más con la edad ^16^, pero son evidentes en pacientes con un año de edad. A nivel corporal es evidente la hipertricosis generalizada, prominentemente a nivel dorsal lo cual se ha reportado en más del 80% de los pacientes ^1^^,^^5^^,^^6^. La hipertricosis cubiti asociada con este síndrome y considerada la característica más prominente no está presente en nuestra paciente, tampoco hay historia de retraso del crecimiento prenatal como han informado otros autores ^1^^,^^3^^,^^5^^,^^6^, no presentó microcefalia como se encuentra en el 50% de los pacientes con SWS ^5^. Las características fenotípicas de nuestra paciente se resumen en la Tabla 1**.**

**Tabla 1:** Resumen de las características fenotípicas presentes en más del 60% de pacientes con SWS.

Características fenotípicas presentes en la paciente	% de pacientes con SWS que presentan la característica
Retardo del crecimiento postnatal	100
Retraso en el desarrollo psicomotor	100
Retardo mental variable	100
Puente nasal deprimido	100
Nariz bulbosa	100
Pestañas largas	94.7
Puente nasal ancho	89.5
Fisuras palpebrales inclinadas hacia abajo	88.9
Cabello grueso	85.7
Almohadillas digitales prominentes	83.3
Hipertricosis dorsal	83.3
Línea de implantación del cabello baja	75.0
Punta nasal ancha	73.7
Cejas gruesas	71.4
Hipertelorismo	66.7
Hipotonía	66.7
Ptosis	63.6
Características fenotípicas no presentes en la paciente	% de pacientes con SWS que presentan la característica
Paladar alto	88.3
Retardo del crecimiento prenatal	66.7
Columela corta	66.7
Macroglosia	66.7
Pliegue palmar único	66.7
Manos y pies pequeños	63.6
Clinodactilia	60.0

Las manifestaciones neurológicas reportadas en SWS incluyen retraso mental y retraso del desarrollo psicomotor en el 100% de los pacientes e hipotonía en el 66.7%, ambas presentes en la paciente, quien además cursa con retraso del desarrollo del habla. No es posible debido a su edad valorar coeficiente intelectual. El comportamiento se caracteriza por irritabilidad sin ser claro un componente de hiperactividad, lo cual se ha reportado en cerca del 20% de los pacientes con este síndrome, quienes cursan con trastornos del comportamiento como heteroagresión, hiperactividad y rasgos autistas ^17^. La paciente cursa además con luxación congénita de la cadera que se ha reportado en el 34% de los pacientes con SWS ^11^. Se descartó mediante estudios otras características reportadas en los pacientes como alteraciones en la alimentación (58% de los pacientes) y otras menos frecuentes como alteraciones del sistema urinario (34.0% de pacientes) y anomalías cardiacas (31.3% de los pacientes) importantes por su asociación con alta tasa de morbilidad ^1^^,^^5^^,^^17^^,^^18^.

El gen afectado KMT2A (lisina metiltransferasa 2A) codifica una histona metiltransferasa relacionada con la regulación de la expresión génica, mediante la modificación de la compactación del DNA en el desarrollo temprano y la hematopoyesis en el proceso de regulación de transcripción mediada por la remodelación de cromatina y se expresa ampliamente en la mayoría de los tejidos humanos ^14^^,^^15^ contiene 36 exones y dos transcritos que codifican diferentes isoformas (Ref Seq 2010). Hasta el momento todas las mutaciones reportadas causantes de WDSTS son *de novo*, y con cada reporte clínico se reporta también generalmente una nueva variante. La mayoría de las mutaciones son de tipo *nonsense, frameshit, splice site* o *exon deletion* las cuales terminan en un transcrito (ARN mensajero) truncado. La mutación aquí reportada c. 4177dupA (p. Ile1393Asn*fs* * 14) no está presente en 1,000 Genomes Project, ExAC, EVSor in-house database, y no ha sido reportada en la literatura. Las mutaciones de tipo frameshift, como esta constituyen un criterio moderado para la clasificación de patogenicidad de variantes de la ACMG ya que modifican la longitud de la proteína, al insertar codones de parada prematuros con el corrimiento del marco de lectura, por lo que normalmente se relacionan con fenotipos severos en enfermedades monogénicas ^10^. 

Las variantes patogénicas del gen KMT2A causantes del SWS se han distribuida a lo largo de todo el gen, todas localizadas antes del dominio FYRC que es importante para el reconocimiento de la proteína posterior al clivaje. Más de la mitad de las mutaciones se han ubicado en el exón 3 y 27, los cuales son los dos exones más largos del gen, sin embargo, no es claro el comportamiento de sitios *hot spot* mutacional ^5^. La paciente aquí reportada tiene una variante en el exón 9, entre dos dominios conservados de esta proteína como lo son el dominio rico en cisteína CXXC homologo a DNA meltil transferasa y el motivo PHD (Plant homeodomain Finger) (Fig. 3) los cuales permiten la unión específica a motivos CpG no metilados del DNA, esencial para el reconocimiento génico, transactivación y transformación asociado a las proteínas MLL ^19^. Existe solo un paciente reportado por Mendelsohn *et al*^20^ con una mutación similar, en ese caso una deleción intragénica que implicaba los exones 2-10 (Tabla 2), el cual llamativamente presentaba infecciones recurrentes del tracto urinario y alteraciones renales. Nuestra paciente ha tenido dos infecciones urinarias, una de ellas complicada con pielonefritis a los 20 meses por lo cual requirió valoración por Nefrología, quienes descartaron alteración renal en las imágenes realizadas, sin embargo, existe una recomendación actual para el seguimiento con Ecografía renal de manera rutinaria en los pacientes con diagnóstico de SWS ^6^. La alteración de la paciente, en estado heterocigoto con herencia autosómica dominante se comporta como un alelo nulo, con una proteína truncada prematuramente que interfiere con la proteína normal y que es similar a lo reportado en la literatura donde normalmente hay un alelo nulo o con pérdida de función con efecto dominante negativo sobre el alelo normal ^6^.

**Figura 3:** **A.** Localización de la mutación en KMT2A reportada. Electroferograma del exón 9 del gen KMT2A que evidencia la duplicación de la Adenina (flecha azul) en la posición 4177 c.4177dupA, la cual tiene un efecto de corrimiento de marco de lectura, generando un codón de parada prematura 13 posiciones más adelante (Recuadro rojo). B. Posición a nivel de la proteína: La mutación causa una terminación prematura de la proteína antes de la traducción de la región c-terminal (Flecha negra) que contiene el dominio BD, dominio de transactivación y dominio SET, así como los motivos PHD esenciales para el reconocimiento génico, transactivación y transformación asociado a la proteína y los motivos FRYN, FYRC y Win.

**Tabla 2:** Resumen de las mutaciones en KMT2A reportadas en pacientes con SWS.

Pacientes	Cambio en el aminoácido	Region afectada del gen	Etnia	Autor
WDST1	p.V2936*	Exón 27	No reportada	Jones et al. ^1^
WDST2	p.L2756*	Exón 27		
WDST3	p.S2305Lfs*2	Exón 27		
WDST5	p.R2382*	Exón 27		
WDST6	p.K1534*	Exón 13		
Paciente 1	p.C1448R	Exón 11	Mexicano	Strom et al. ^7^
Paciente 2	c.4086+1G>A	Intron 8	Caucásico	
	chr11:118,339,487-118,355,089del	Exón 2 to 10	Hispánico	Mendelson et al. ^20^
P1	p.G2422*	Exón 27	No reportada	Zemojtel et al.^21^
P8	p.E3448fs*7	Exón 27		
	p.R2127*	Exón 26	No reportada	Calvel et al. ^22^
Gemelo 1	p.R1083*	Exón 4	Caucásico	Dunkerton et al. ^18^
Gemelo 2	p.R1083*	Exón 4		
	p.R1636*	Exón 15	Árabe	Steel et al. ^23^
Paciente 1	p.R2480*	Exón 27	Japonés	Miyake et al. ^24^
Paciente 2	p.Q2261*	Exón 27	Japonés	
Paciente 3	p.C1189Y	Exón 5	Australiano	
Paciente 4	p.Pro280Thr	Exón 3	Japonés	
Paciente 5	p.V347Lfs*53	Exón 3	Japonés	
Paciente 6	p.L717Cfs*39	Exon 3	Japonés	
CdLS 3	p.R745*	Exón 3	Turco	Yuan et al. ^25^
	p.C1161G	Exón 5	Italiano	Stellacci et al. ^26^
A.II-5	p.Q2803*	Exón 27	Chino	Sun et al. (5)
B.II-1	p.Q819*	Exón 3	Chino	
	p. Pro51Argfs*84	Exón 1	No reportada	Argawall et al. ^27^
	p. Ile1393Asnfs*14	Exón 9	Colombiano	Este reporte

En conclusión, se reporta por primera vez una paciente de origen colombiano con una variante patogénica de tipo frameshift no reportada en KMT2A. Sus características son similares fenotípicamente a lo reportado en la literatura, con una historia clínica de episodios de infección urinaria sin alteración renal, lo cual parece relacionarse con la actividad metil transferasa de la proteína ubicada en los dominios afectados por la mutación aquí reportada. Esta sería una aproximación a la correlación genotipo-fenotipo que se propone para el SWS, donde además de la hipertricosis se debe analizar el compromiso neurológico y renal. Las características más prominentes del SWS que pueden servir como criterio diagnóstico son: hipertricosis generalizada, retardo del crecimiento postnatal, retraso del desarrollo psicomotor y fenotipo facial distintivo (cabello y cejas gruesas, hipertelorismo, fisuras palpebrales inclinadas hacia abajo y pestañas largas). Estas características están presentes en más del 70% de los pacientes reportados en la literatura mundial.
